# Observations on Cold Water in Gout

**Published:** 1808-06

**Authors:** 


### *To the Editors of the Medical and Physical Journal.*

Gentlemen,

THROUGH the medium of your widely extended and useful Journal, I wish to address a few words to Dr. Kinglake, on the subject of the mode of treatment (which has lately been revived by him) in the Gout. A practice which I am induced to believe, from my own limited experience, may, in a great many instances, be safely and beneficially employed. The few cases (six in number) wherein I have had recourse to the refrigerating plan, were all subjects in high health, in the meridian of life, and who had only experienced one or two attacks of the disease; ease; and where it was entirely confined to the first joint of the great toe. The application of cold (simply water) was preceded by the free use of leeches, which part of the practice I consider absolutely necessary, by unloading the surrounding parts, and thus giving the distended vessels a better opportunity of recovering their original state. These cases all terminated to the entire satisfaction of myself and patients.

Three only have had any relapse, and these were brought on by over exertion in walking and free living. The same treatment was again pursued in each, without any reference to me, and with the most complete success.

In my opinion this is the only form of the disease, and the only kind of subjects, wherein this mode of treatment can with propriety be adopted. It will be only a waste of time to endeavour to point out the varieties of the complaint, wherein this remedy is not applicable: they may be enumerated in a few words. In old and debilitated subjects, or where the disease shews any irregularity in its attacks, or where the head, stomach, or any other parts, beside the extremities have suffered, or lastly, where the patient has had repeated and severe attacks of the disorder, leaving behind contracted and stiffened joints. These subjects I consider as running great risque by the external application of cold in any form.

The good opinion I have hitherto entertained of the propriety of the cold plan, even in recent cases, has I must confess been somewhat diminished, by what happened in the following case. As I never saw an instance of the disease terminating in the same way before; and as I am totally at a loss how to account for it, I have taken the liberty of addressing Dr. K. on the subject; being convinced, from the candour and manliness he has uniformly maintained throughout the important investigation he has been so long engaged in, that he will, with his usual ingenuity, explain to me, a phaenomenon, which, as I before remarked, has never come under my own observation.

Mr. S. a vintner, aged 41, originally brought up a cabinet-maker, of regular habits, and very industrious, one night was awoke with a burning pain in the ball of the great toe, which kept him awake the remainder of the night. In the morning a considerable degree of tumefaction and redness had taken place, attended with much pain, and a total inability of motion. The system was in some measure affected, with a whitish tongue, the skin hot, and the pulse quick. Upon inquiry, this was the first attack; tack of the kind he had ever experienced. His father and an uncle had been subject to Gout for many years. An opening draught was directed to be taken immediately, six leeches applied to the part affected, and the bleeding encouraged by flannels loaded with vapour from warm water, applied as long as the blood would flow; after which the whole foot was to be kept immersed in cold water, or covered with folded linen moistened with the same.

In a few hours the pain was considerably relieved, and the following night passed over without any material disturbance. Three days afterwards, by pursuing the same means, he was free from lameness, and able to follow his ordinary avocations. During this short confinement, his diet was strictly antiphlogistic, and the bowels regularly opened by the daily exhibition of an opening draught. On the following day, he assisted in removing a heavy cask from the cellar, when he was suddenly seized with a violent pain in the elbow: this he considered at the time, as the effects of over exertion; but the increased violence of the pain, accompanied with a great degree of swelling and redness, soon convinced him of the resemblance it bore to the attack he had so lately experienced in the great toe. Under this impression, of his own accord, he had immediate recourse to those means, which had already afforded him so much relief. The cold water was applied freely, and an opening medicine taken, but the application of leeches was omitted. In 12 hours the pain in the elbow was relieved, but the two first fingers of the hand of the same side were swollen, red, and painful. Twenty-four hours after the first application of cold water to the elbow, I found him extremely uncomfortable, the tongue white, pulse quick, with a great degree of irritation over the whole system. Pills composed of antimonial powder, calomel, and opium, were directed to be taken every four hours. The pediluvium with warm vinegar and water were also employed. These means produced some abatement in the pain, and the tension of the elbow and fingers was considerably reduced: still a degree of incoherency in his conversation continued, which I would willingly have ascribed to the effects of opium. This delusion could not, however, be for a moment admitted. The pulse was tremulous, the countenance expressive of great anxiety, and the whole body bedewed with a cold clammy sweat. My attention for the first time was now directed by the nurse to a pain he complained of just above the pubis, accompanied with a difficulty of making water.

On

On examination I was very much astonished to find the penis and scrotum excessively tumefied, and of a livid color, putting on the appearance these parts have, when filled with urine in cases of ulcerated or ruptured urethra.

On inquiry I found this change had began to take place the day before; the difficulty in voiding the urine merely arose from the tumefied state of the prepuce, impeding the free flow from the orifice of the urethra. Could this threatning of sphacelus have arisen from a metastasis of gouty matter? Was gout ever known to have attacked these parts, beyond that of occasionally causing a slight degree of ardor urinæ, accompanied with a discharge of purulent mucus from the urethra? Whatever might have been the exciting cause, the proper mode of treatment was obvious. Cinchona and opium were largely administered, and the fermenting cataplasm applied to the parts affected: Wine and other cordials were also directed to be given in as great quantities as the patient thought proper to take. In 16 hours the disease put on a more favourable appearance; the livid colour disappeared, and the tumefaction began to subside; his recovery was uninterrupted, and the patient is at this time in a state of complete convalescence. I should mention, that during the progress of his recovery he did not experience gouty pains in any other part.

VERAX.

*London, May* 1, 1808.

